# Supplementary material for: Content-rich biological network constructed by mining PubMed abstracts
Source: BMC Bioinformatics. 2004 Oct 8;5:147. doi: 10.1186/1471-2105-5-147 (PMC528731; doi:10.1186/1471-2105-5-147)
Supplement: Additional File 2 — The original results of the above study (non-essential files are deleted to keep the file size under the limit set by BMC bioinformatics). [file 1471-2105-5-147-S2.bz2 › chilibotAdditionalFile2/dip50/471ID9660793E10527/html/CCL5_CXCR3.html]

 


 **CCL5** and **CXCR3** 
  
Found 237 abstracts in PubMed, retrieved 50.  
 

 What does Google say? 
 PDF only 
| .edu only 

---

**Interactive relationship** (e.g. stimulation, inhibition, etc)

**Neutral relationship**- BACKGROUND Interactions between chemokines IP 10  [ **CXCR3** ] , MCP 1, and RANTES  [ **CCL5** ]  and their receptors may mediate graft rejection following islet transplantation.  Ref: 12697414 J Surg Res, 2003
- The present findings suggest that MCP 1, RANTES  [ **CCL5** ] , and IP 10  [ **CXCR3** ]  may participate in the pathogenesis of mercuric chloride induced tubulointerstitial fibrosis with mononuclear cell infiltration, via CCR2, CCR1 or CCR5, and  **CXCR3** , respectively.  Ref: 12834626 Exp Mol Pathol, 2003
- DESIGN AND PATIENTS Immunocytochemistry and reverse transcriptase polymerase chain reaction RT PCR amplification were used to analyse the expression of monocyte chemoattractant protein MCP 1, RANTES  [ **CCL5** ]  regulated upon activation, normal T cell expressed and secreted, macrophage inflammatory protein MIP 1alpha, MIP 1beta, interferon IFN gamma inducible protein IP 10  [ **CXCR3** ]  and monokine induced by IFN gamma Mig in thyroid tissue from patients with Hashimoto s thyroiditis n = 4, Graves disease n = 6 and nonautoimmune multinodular goitre n = 4.  Ref: 12864798 Clin Endocrinol (Oxf), 2003
- Xenograft rejection was associated with early intragraft transcript expression for monocyte chemotactic protein 1 MCP 1 3 to 5 days, IP 10  [ **CXCR3** ]  3 to 4 days and macrophage inflammatory protein 1alpha MIP 1alpha 3 to 5 days and subsequent expression of eotaxin days 4 to 10, MIP 1beta days 4 and 5 and regulated on activation, normal T cell expressed and secreted RANTES  [ **CCL5** ]  days 4 to 6 mRNA.  Ref: 12588649 Xenotransplantation, 2003

**Non-interactive relationship** (e.g. studied together, co-existance, homology, etc.)

- We therefore compared intracellular RANTES  [ **CCL5** ]  and IP 10  [ **CXCR3** ]  production as well as CCR5,  **CXCR3** , and CXCR1 expression by gamma delta T cells derived from the blood and CSF of patients with MS and healthy controls HC.  Ref: 12173306 Clin Immunol, 2002
- S. aureus infection also results in secretion of these chemokines but secretion is delayed and of lesser magnitude 210 10 pg ml per 4 x 10 5 cells, 1 70 1240 pg ml per 4 x 10 5 cells, 930 34 pg ml per 4 x 10 5 cells, and 1 70 720 pg ml per 4 x 10 5 cells for IL 8, IP 10  [ **CXCR3** ] , RANTES  [ **CCL5** ] , and MCP 1, respectively.  Ref: 12211439 J Bone Miner Res, 2002
- RESULTS Expression of IP 10  [ **CXCR3** ] , Mig, I TAC, RANTES  [ **CCL5** ] ,  **CXCR3** , and CCR5, but not MCP 1 and IL 8, increased significantly in both grade 2 and grade 3 rejection P< ; =0.0096.  Ref: 12544874 Transplantation, 2003
- Increased levels of CXCL10 IP 10  [ **CXCR3** ]  and  **CCL5**  RANTES  [ **CCL5** ]  in cerebrospinal fluid were also observed in a subgroup of MS patients.  Ref: 12356205 Mult Scler, 2002
- However, the expression of macrophage inflammatory protein MIP 1alpha, RANTES  [ **CCL5** ]  regulated on activation normal T cells expressed and secreted, and interferon gamma inducible protein 10 IP 10  [ **CXCR3** ]  was not changed in HT 29 or Caco 2 cells stimulated with toxin A.  Ref: 12139393 Microbiol Immunol, 2002
- Potential molecular differences between early and late rejection grade > =2 episodes were examined by testing chemokine and receptor gene expression.METHODS Expression of inducible protein IP 10  [ **CXCR3** ] , monokine induced by IFN gamma Mig, interferon inducible T cell alpha chemoattractant I TAC, regulated on activation normal T cell expressed and secreted RANTES  [ **CCL5** ] , and their receptors  **CXCR3**  and CCR5 was tested in 60 endomyocardial biopsies from 24 patients using quantitative Taqman real time polymerase chain reaction PCR.  Ref: 12829909 Transplantation, 2003
- Finally, TNFalpha IFNgamma was the most significant cytokine combination in leading to a pronounced upregulation in a comparable, time dependent manner of the production of chemokines IP 10  [ **CXCR3** ]  CXCL10, RANTES  [ **CCL5** ]   **CCL5** , MIG CXCL9, MCP 1 CCL2, and IL 8 CXCL8.  Ref: 12555203 Glia, 2003
- However, the mRNA expressions of macrophage inflammatory protein 1alpha MIP 1alpha, MIP 1beta, interferon inducible protein 10 IP 10  [ **CXCR3** ] , RANTES  [ **CCL5** ] , lymphotactin and I 309 were either very weak or undetectable.  Ref: 12402381 Yonsei Med J, 2002
- Despite there being no difference in expression of CC chemokine receptor 3  [ **CXCR3** ] , the priming effect of RANTES  [ **CCL5** ]  and eotaxin was significantly enhanced in eosinophils from the patients.  Ref: 12797483 Eur Respir J, 2003
- CONCLUSION We postulate that TFC may play a role in the pathogenesis of autoimmune thyroid disease as they are able to express the chemokines MIP 1alpha, MIP 1beta, MCP 1, RANTES  [ **CCL5** ] , IP 10  [ **CXCR3** ]  and Mig that would promote the infiltration of immune cells into the thyroid gland.  Ref: 12864798 Clin Endocrinol (Oxf), 2003
- RESULTS AND DISCUSSION Cross talk between RS cells and reactive elements involves several cytokine chemokines that process proliferative interleukin IL 13 and IL 17, immunosuppressive IL 10 and transforming growth factor beta and background formation IL 5, TARC, MDC, IP 10  [ **CXCR3** ] , RANTES  [ **CCL5** ] , Mig and others messages.  Ref: 12078904 Ann Oncol, 2002
- Infiltrating CD8 T Cells in Oral Lichen Planus Predominantly Express CCR5 and  **CXCR3**  and Carry Respective Chemokine Ligands RANTES  [ **CCL5** ]   **CCL5**  and IP 10  [ **CXCR3** ]  CXCL10 in Their Cytolytic Granules A Potential Self Recruiting Mechanism.  Ref: 12819030 Am J Pathol, 2003
- Three of the genes overexpressed by ALR tumors were the chemokines small inducible cytokine A4 MIP 1beta, RANTES  [ **CCL5** ] , and interferon inducible protein 10 IP 10  [ **CXCR3** ] .  Ref: 12473593 Clin Cancer Res, 2002
- Furthermore, infiltrating T cells, particularly CD8 T cells, were positive for RANTES  [ **CCL5** ]   **CCL5**  and IP 10  [ **CXCR3** ]  CXCL10, the ligands of CCR5 and  **CXCR3** , respectively.  Ref: 12819030 Am J Pathol, 2003
- We investigated the potential down regulators of chemokine secretion IL 4, IL 10, IL 13, dexamethasone, and PGE2, IL 10 and IL 13 down regulate M. tuberculosis induced IL 8, IP 10  [ **CXCR3** ] , RANTES  [ **CCL5** ] , and MCP 1 secretion from MG 63 cells by between 48 and 94% P <.  Ref: 12797540 J Clin Immunol, 2003
- This pattern was consistent with the early recruitment of macrophages MCP 1, MIP 1alpha, the influx of CD4 T cells MCP 1, MIP 1alpha, MIP 1beta, IP 10  [ **CXCR3** ]  and RANTES  [ **CCL5** ]  and the characteristic infiltrate of eosinophils eotaxin and RANTES  [ **CCL5** ]  associated with islet xenograft rejection.  Ref: 12588649 Xenotransplantation, 2003
- The presence of chemokine MCP 1, MIP 1alpha, MIP 1beta, IP 10  [ **CXCR3** ] , RANTES  [ **CCL5** ]  positive cells and chemokine receptor CCR5,  **CXCR3**  positive cells in inflamed human gingival tissues.  Ref: 12445801 Cytokine, 2002
- Proinflammatory and type 1 cytokines including TNF alpha, IL 1beta, IFN gamma, IL 2, RANTES  [ **CCL5** ] , and IP 10  [ **CXCR3** ]  were elevated in CNS tissues from mice with PREMO.  Ref: 12486156 J Neurosci, 2002
- Chemokines such as interleukin 8 IL 8, RANTES  [ **CCL5** ] , IP 10  [ **CXCR3** ] , MIG or lymphotactin, do not appear to induce fibrosis when over expressed in rodent lung.  Ref: 12570673 Curr Pharm Des, 2003
- The PBMC levels of CCR5 ligands MIP 1 alpha beta and RANTES  [ **CCL5** ] , and chemokines MCP 1, IL 8, lymphotactin, IP 10  [ **CXCR3** ]  and I 309 were analysed by ribonuclease protection assay RPA.  Ref: 12021948 J Neurol, 2002
- Modification with live but not inactive NDV induced in all human tumor cells IFN beta and the chemokines RANTES  [ **CCL5** ]  and IFN gamma inducible protein 10 IP 10  [ **CXCR3** ] .  Ref: 12063554 Int J Oncol, 2002
- RT PCR and RNase Protection Assay were used to measure mRNA expression for the following chemokines IP 10  [ **CXCR3** ] , MIP 1alpha, MIP 1beta, MCP 1, and RANTES  [ **CCL5** ] .  Ref: 12697414 J Surg Res, 2003
- The CXC chemokines such as IL 8, IP10  [ **CXCR3** ] , Mig, SDF 1 alpha or CC chemokines such as MCP 1, MIP 1 alpha, eotaxin, RANTES  [ **CCL5** ]  have been frequently harvested from tumor tissues or the biological fluids of patients.  Ref: 12489485 Recenti Prog Med, 2002
- ELISA showed high levels of the chemokine RANTES  [ **CCL5** ]   **CCL5**  for CCR5 and IP 10  [ **CXCR3** ]  CXCL10 for  **CXCR3**  in cytokine stimulated PTEC supernatant.  Ref: 11981057 Nephrol Dial Transplant, 2002
- CONCLUSION These results suggest that RANTES  [ **CCL5** ] , IP 10  [ **CXCR3** ]  and MCP 1 may contribute to the recruitment of Th1 type T cells into the eye during the development of EAU in mice.  Ref: 12605265 Graefes Arch Clin Exp Ophthalmol, 2003
- RESULTS The gene expression of RANTES  [ **CCL5** ] , IP 10  [ **CXCR3** ] , and MCP 1 was upregulated on day 14 after immunization early stage of EAU.  Ref: 12605265 Graefes Arch Clin Exp Ophthalmol, 2003
- From among the 13 chemokines examined, MCP 3, MCP 4, RANTES  [ **CCL5** ] , and IP 10  [ **CXCR3** ]  were detected in the disc from the idiopathic scoliosis, and MCP 3, MCP 4, RANTES  [ **CCL5** ] , IP 10  [ **CXCR3** ] , MIG, and MGSA alpha were detected in the infected or herniated discs.  Ref: 12131709 Spine, 2002
- IP 10  [ **CXCR3** ] , but not RANTES  [ **CCL5** ] , is upregulated by leptin in monocytic cells.  Ref: 12668159 Cytokine, 2003
- The expression of the genes encoding chemokines cytokines was dramatically changed, such as Mig, IP 10  [ **CXCR3** ] , RANTES  [ **CCL5** ] , TNF alpha, and IFN gamma.  Ref: 12419307 Biochem Biophys Res Commun, 2002
- RESULTS Alloantigen primed T cells induced endothelial cell expression of regulated on activation normal T cell expressed and secreted RANTES  [ **CCL5** ] , IP 10  [ **CXCR3** ] , Mig, monocyte chemotactic protein 1, macrophage inflammatory protein 1alpha, and macrophage inflammatory protein 1beta within 5 hr of coculture.  Ref: 12605122 Transplantation, 2003
- NO donors diminished in a dose dependent manner and at both mRNA and protein levels the IP 10  [ **CXCR3** ] , RANTES  [ **CCL5** ] , and MCP 1 expression in keratinocytes cultured from healthy patients and psoriatic patients.  Ref: 12368213 Am J Pathol, 2002
- GS NO treated psoriatic skin showed reduction of IP 10  [ **CXCR3** ] , RANTES  [ **CCL5** ] , and MCP 1, but not interleukin 8 expression by keratinocytes.  Ref: 12368213 Am J Pathol, 2002
- Increased expression of the chemokine receptors CCR5 and  **CXCR3**  on T cells and raised amounts of the chemokines RANTES  [ **CCL5** ]  and IP 10  [ **CXCR3** ]  have been noted in the CSF and brain tissue of MS patients, but the contribution of gamma delta T cells to these increases is unknown.  Ref: 12173306 Clin Immunol, 2002
- Amplified fragments corresponding to KC, IP10  [ **CXCR3** ] , Mig 1, BCL, MIP 2, SLC, RANTES  [ **CCL5** ] , MCP 1, C10, and Lptn were obtained, and confirmed by Southern blot and sequencing.  Ref: 12667214 Immunology, 2003
- CONCLUSION The expression of IP 10  [ **CXCR3** ] , Mig, I TAC, RANTES  [ **CCL5** ] ,  **CXCR3** , and CCR5 in cardiac allografts significantly correlates with the presence and grade of acute rejection.  Ref: 12544874 Transplantation, 2003
- Furthermore, the presence of  **CCL5**  and CXCL10 in the cytolytic granules of tissue infiltrating CD8 T cells expressing CCR5 and  **CXCR3**  reveals a potential self recruiting mechanism involving activated effector cytotoxic T cells.  Ref: 12819030 Am J Pathol, 2003
- We show that M. tuberculosis infection of cultured and primary osteoblasts induces extensive secretion of the chemokines interleukin IL 8, inducible protein IP 10  [ **CXCR3** ] , RANTES  [ **CCL5** ] , and monocyte chemoattractant protein MCP 1 within 72 h 1630 280 pg ml per 4 x 10 5 cells, 7 30 8480 pg ml per 4 x 10 5 cells, 1 30 3040 pg ml per 4 x 10 5 cells, and 13 70 1 40 pg ml per 4 x 10 5 cells, respectively, for MG 63 osteoblasts.  Ref: 12211439 J Bone Miner Res, 2002
- The RNA was reverse transcribed, and the resultant cDNA was amplified by PCR using primer pairs specific to the CXC chemokines IL 8, MGSA alpha, IP 10  [ **CXCR3** ] , MIG, the CC chemokines MCP 1, MCP 2, MCP 3, MCP 4, MIP 1alpha, MIP 3alpha, RANTES  [ **CCL5** ] , STCP 1, the C chemokine lymphotactin, and the glyceraldehyde phosphate housekeeping gene.  Ref: 12131709 Spine, 2002
- IL 16, IL 8, IP 10  [ **CXCR3** ]  and RANTES  [ **CCL5** ]  are participated in the accumulation of CD4 T cell.  Ref: 12233067 Nippon Rinsho, 2002
- Alloantigen primed CD4 T cells induced endothelial expression of IP 10  [ **CXCR3** ]  and RANTES  [ **CCL5** ]  but none of the other chemokines tested.  Ref: 12605122 Transplantation, 2003
- RESULTS Expression of MCP 1, RANTES  [ **CCL5** ] , MIP 1alpha, MIP 1beta, IP 10  [ **CXCR3** ]  and Mig was demonstrated in all Hashimoto s and most Graves thyroid specimens but very little expression was detected in the nonautoimmune goitre samples.  Ref: 12864798 Clin Endocrinol (Oxf), 2003
- In the lesions, the expression of MCP 1 mRNA was most prominently elevated, and those of RANTES  [ **CCL5** ]  and IP 10  [ **CXCR3** ]  mRNAs also increased, and their proteins were localized in tubular epithelium.  Ref: 12834626 Exp Mol Pathol, 2003
- We found no significant changes in constitutive RANTES  [ **CCL5** ]  and inducible IP 10  [ **CXCR3** ]  production following LP MW irradiation.  Ref: 12669299 Bioelectromagnetics, 2003
- High levels of RANTES  [ **CCL5** ]   **CCL5** , MCP 1 CCL2, IP 10  [ **CXCR3** ]  CXCL10, and Mig CXCL9 simultaneously with mononuclear cell infiltration in the lungs was found.  Ref: 12875978 Am J Pathol, 2003
- The expression of interferon IFN gamma inducible protein IP 10  [ **CXCR3** ] , monokine induced by IFN gamma Mig, interferon inducible T cell alpha chemoattractant I TAC, regulated on activation normal T cell expressed and secreted RANTES  [ **CCL5** ] , monocyte chemotactic protein MCP 1, interleukin IL 8, and the receptors  **CXCR3**  and CCR5 were tested using quantitative, real time polymerase chain reaction.  Ref: 12544874 Transplantation, 2003
- CONCLUSIONS This study suggested diagnostic as well as potential functional roles of the chemokine receptor pairs IP10  [ **CXCR3** ]   **CXCR3** , RANTES  [ **CCL5** ]  CCR5, and MCP1 CCR2 in rat models of transplant vasculopathy.  Ref: 12398874 J Heart Lung Transplant, 2002
- In this study we analyzed whether LP MW irradiation modulated the production of chemokines, including RANTES  [ **CCL5** ]  and IP 10  [ **CXCR3** ]  of keratinocytes in vitro.  Ref: 12669299 Bioelectromagnetics, 2003
- Macrophage chemotactic protein 1 MCP 1, macrophage inflammatory protein MIP 1alpha, MIP 1beta, IFN gamma inducible protein 10 IP 10  [ **CXCR3** ]  and RANTES  [ **CCL5** ]  producing cells were found to be present in inflamed human gingival tissues.  Ref: 12445801 Cytokine, 2002
- Neuronal and astrocytic apoptosis in these cultures was significantly inhibited by co treatment with MCP 1 or RANTES  [ **CCL5** ]  but not IP 10  [ **CXCR3** ] .  Ref: 12753088 J Neurochem, 2003
- The biopsies were taken in the first 3 months or from the 9th to the 12th month following transplantation.RESULTS IP 10  [ **CXCR3** ] , Mig, RANTES  [ **CCL5** ] ,  **CXCR3** , and CCR5 expression levels were increased in the later versus earlier biopsies P< ; =0.01 despite no change in histologic rejection grade status.CONCLUSION These results demonstrate significantly increased expression of T cell chemoattractants in heart allografts during later rejection when compared with episodes occurring shortly after transplantation.  Ref: 12829909 Transplantation, 2003
- Interestingly, these in vivo effects on tumor growth correlated with higher mRNA expression of chemokines such as MIP 1beta, RANTES  [ **CCL5** ] , and IP 10  [ **CXCR3** ] , as well as dramatic increases in vitro in the inducibility of cytokine mRNA such as IFN beta, TNF alpha and interleukin 6.  Ref: 12234977 Cancer Res, 2002
